# Supplementary material for: The assessment of psychometric properties of childbirth violence questionnaire in Iranian women
Source: PLoS One. 2025 Sep 4;20(9):e0328932. doi: 10.1371/journal.pone.0328932 (PMC12410820; doi:10.1371/journal.pone.0328932)
Supplement: Attachment — Submitted filename: Response to Reviewers.doc [file pone.0328932.s002.doc]

ONE-D-24-48647

Development and Psychometric Childbirth Violence Questionnaire in Iranian Women

PLOS ONE

Dear Dr. direkvand-moghadam,

Thank you for submitting your manuscript to PLOS ONE. After careful consideration, we feel that it has merit but does not fully meet PLOS ONE’s publication criteria as it currently stands. Therefore, we invite you to submit a revised version of the manuscript that addresses the points raised during the review process.

Please submit your revised manuscript by Mar 17 2025 11:59PM. If you will need more time than this to complete your revisions, please reply to this message or contact the journal office at [plosone@plos.org](mailto:plosone@plos.org). When you're ready to submit your revision, log on to https://www.editorialmanager.com/pone/ and select the 'Submissions Needing Revision' folder to locate your manuscript file.

Please include the following items when submitting your revised manuscript:

- A rebuttal letter that responds to each point raised by the academic editor and reviewer(s). You should upload this letter as a separate file labeled 'Response to Reviewers'.
- A marked-up copy of your manuscript that highlights changes made to the original version. You should upload this as a separate file labeled 'Revised Manuscript with Track Changes'.
- An unmarked version of your revised paper without tracked changes. You should upload this as a separate file labeled 'Manuscript'.

If you would like to make changes to your financial disclosure, please include your updated statement in your cover letter. Guidelines for resubmitting your figure files are available below the reviewer comments at the end of this letter.

If applicable, we recommend that you deposit your laboratory protocols in protocols.io to enhance the reproducibility of your results. Protocols.io assigns your protocol its own identifier (DOI) so that it can be cited independently in the future. For instructions see: [https://journals.plos.org/plosone/s/submission-guidelines#loc-laboratory-protocols](https://journals.plos.org/plosone/s/submission-guidelines#loc-laboratory-protocols). Additionally, PLOS ONE offers an option for publishing peer-reviewed Lab Protocol articles, which describe protocols hosted on protocols.io. Read more information on sharing protocols at <https://plos.org/protocols?utm_medium=editorial-email&utm_source=authorletters&utm_campaign=protocols>.

We look forward to receiving your revised manuscript.

Kind regards,

Othman A. Alfuqaha, Ph.D.
Academic Editor
PLOS ONE

Journal Requirements:

When submitting your revision, we need you to address these additional requirements.

1. Please ensure that your manuscript meets PLOS ONE's style requirements, including those for file naming. The PLOS ONE style templates can be found at

https://journals.plos.org/plosone/s/file?id=wjVg/PLOSOne_formatting_sample_main_body.pdf and

<https://journals.plos.org/plosone/s/file?id=ba62/PLOSOne_formatting_sample_title_authors_affiliations.pdf>

2. Please provide additional details regarding participant consent. In the ethics statement in the Methods and online submission information, please ensure that you have specified what type you obtained (for instance, written or verbal, and if verbal, how it was documented and witnessed). If your study included minors, state whether you obtained consent from parents or guardians. If the need for consent was waived by the ethics committee, please include this information.

Once you have amended this/these statement(s) in the Methods section of the manuscript, please add the same text to the “Ethics Statement” field of the submission form (via “Edit Submission”).

For additional information about PLOS ONE ethical requirements for human subjects research, please refer to [http://journals.plos.org/plosone/s/submission-guidelines#loc-human-subjects-research](http://journals.plos.org/plosone/s/submission-guidelines#loc-human-subjects-research).

3. Please provide a complete Data Availability Statement in the submission form, ensuring you include all necessary access information or a reason for why you are unable to make your data freely accessible. If your research concerns only data provided within your submission, please write "All data are in the manuscript and/or supporting information files" as your Data Availability Statement.

4. Your ethics statement should only appear in the Methods section of your manuscript. If your ethics statement is written in any section besides the Methods, please delete it from any other section.

Additional Editor Comments:

After careful evaluations, the reviewers have provided detail feedback on your work. Please answer both reviewer's comments and my comments as follows:

Abstract:
-a mixed-methods approach to evaluate. Which mixed method?

**Authors: This suggestion was made by adding the abstract Methods section**.

-We need sample size and data collection time.

**Authors: Given that the study was conducted in two qualitative and quantitative sections, the number of participants in each section is specified in the text.**

-The CBVQ is a unique tool to assess childbirth violence. Is this sentence correct?

- give us a broader implications.

Authors: The Childbirth Violence Questionnaire (CBVQ) stands out as a unique tool for several reasons:

1. The CBVQ is specifically designed to reflect the experiences and understanding of childbirth violence among Iranian women. It takes into account the cultural and social norms that influence women's perceptions of violence during childbirth, making it more relevant than generic tools that may not consider these factors.
2. Unlike existing tools that primarily focus on the interpersonal relationship between the care team and the mother, the CBVQ encompasses a broader perspective. It includes dimensions of violence from the care team, the health system, and family support, thus providing a more holistic view of the factors contributing to childbirth violence.
3. psychological and physical abuse, non-professional care, delay in offering care, and lack of family support. This multidimensional approach allows for a thorough assessment of various aspects of childbirth violence, which is crucial for understanding its complexity.
4. The CBVQ was developed through a rigorous mixed methods approach, starting with qualitative interviews that informed the creation of the questionnaire. This inductive process ensures that the tool is grounded in the real experiences of women, rather than being a mere adaptation of existing instruments

Introduction
-Your paper needs proofreading by a native English speaker.

Authors: The article was referred to someone who was fluent in English and spelling and grammar corrections were made as much as possible.
- Merge sentence in each other. Paragraphs are too small.

Authors: Several related paragraphs were merged.
- We need something that we dont know in each paragraph.
- Lack of references are noted in this section.
- Please why your scale is important.

Authors: A few paragraphs on the importance of this tool are provided in response to the esteemed referee's question titled:” The CBVQ is a unique tool to assess childbirth violence. Is this sentence correct?

- give us a broader implications.”

- The aim is not clearly mention. Please re-write it again.

Authors: Added at the end of the introduction

Method are critical and need strong revision. For example, data are too old 2019, Your justification?, study design, answer 5 w questions (When, how, where,.....),

Authors: Key Details About answer 5 w questions

When was the study conducted?

The date of the study is provided in the first line of method section.

How was the questionnaire developed?

The questionnaire was developed using a mixed-methods approach, which included qualitative and quantitative phases. Initially, 99 items were identified based on interviews with participants. This was refined to a preliminary questionnaire with 65 items for psychometric testing. The final version, known as the Childbirth Violence Questionnaire (CBVQ), consists of 34 items and was validated through exploratory factor analysis (EFA) and content validity assessments

Where was the study conducted?

The study was conducted in Iran, focusing specifically on the experiences of women during childbirth within the Iranian healthcare system. The cultural and social context of Iran was taken into account while designing the questionnaire to ensure its relevance and applicability

What were the main findings regarding the factors of childbirth violence?

The exploratory factor analysis revealed four main factors related to childbirth violence: psychological and physical abuse, non-professional care, delay in offering care, and lack of family support. These factors explained a total of 63.276% of the variance in the responses, indicating significant areas of concern for women during childbirth

Who were the experts involved in validating the questionnaire?

The validation of the questionnaire involved a panel of experts, including faculty members with expertise in midwifery, reproductive health, psychology, sociology, and nursing. Their feedback was crucial in assessing the content validity of the items included in the questionnaire

-unnecessary information and details, 205 participants how?, analysis section is missing.

Authors: The study involved a specific sample size during its evaluation phases:

Initial Sample for Reliability Testing: The reliability of the tool was assessed using a sample of 30 mothers. This sample was used to calculate internal consistency through Cronbach's alpha coefficient, ensuring that each item correlated well with the overall instrument.

Test-Retest Method: To further evaluate the reliability, the same group of 30 participants completed the questionnaire again under identical conditions after a period of 14 days. This method helped assess the stability of the instrument over time.

Exploratory Factor Analysis (EFA): The Kaiser-Meyer-Olkin (KMO) index was utilized in the study to assess the suitability of the data for factor analysis, which is a critical step in validating the Childbirth Violence Questionnaire (CBVQ). Here are the key reasons for its use:

The KMO index measures the adequacy of the sample size for conducting factor analysis. A higher KMO value indicates that the sample is appropriate for identifying underlying relationships among variables. This is essential for ensuring that the factor analysis yields reliable and valid results.

The KMO index helps determine whether the data is suitable for factor analysis by evaluating the correlations between variables. A KMO value closer to 1 suggests that the data is suitable for factor analysis, while a value below 0.5 indicates that the data may not be suitable. In the present study Kaiser-Meyer-Olkin Measure of Sampling Adequacy was: 0. 777.

The KMO index is often used alongside Bartlett's Test of Sphericity, which tests the hypothesis that the correlation matrix is an identity matrix. Together, these tests provide a comprehensive assessment of whether the data is appropriate for factor analysis. Based on our results KMO P- value = 0.000.

Results
- Start with your demographic factors.

Authors: Because the study was conducted in two phases, the researchers presented the results based on the stages of study design and achievement of the results.
- Tables are too long and unnecessary. Appendix

Authors: We have presented the results and tables for easier understanding for the readers. Some tables have been moved to the appendix section based on the opinion of the referees and the respected editor.
- 0.000 is wrong it should be 0.001.

Authors: Changed in Table 3.

- All results are difficult to read. Please arrange your Tables in 2 Tables for example, mention your results in a scientific way.

Authors: We have presented the results and tables for easier understanding for the readers. Some tables have been moved to the appendix section based on the opinion of the referees and the respected editor.

Discussion
- Do not repeat your goals, highlighted your findings.

Authors: Extra sections removed.

- Discuss your findings with previous studies.

Authors: Some changes were made in the discussion.
- How we can use this scale globally?

Authors: The CBVQ is created for Iranian women's cultural and social context but can be adapted for different cultures. Researchers can change the language and context to fit local norms while keeping the main ideas of the questionnaire. This flexibility is important for its use in various populations.

The scale supports cross-cultural studies on childbirth violence, allowing comparisons between experiences in different countries. Findings can help governments and health organizations create policies to reduce childbirth violence. It can also train healthcare providers to better recognize and address these issues.

- More international studies and references are needed.

Authors: Several international references have been added.
- no need to write strength and limitation in one section.

Authors: Changes applied.
Please take my comments under consideration, otherwise I will reject the paper.

-
-


[Note: HTML markup is below. Please do not edit.]

Reviewers' comments:

Reviewer's Responses to Questions

**Comments to the Author**

1. Is the manuscript technically sound, and do the data support the conclusions?

The manuscript must describe a technically sound piece of scientific research with data that supports the conclusions. Experiments must have been conducted rigorously, with appropriate controls, replication, and sample sizes. The conclusions must be drawn appropriately based on the data presented.

Reviewer #1: No

Reviewer #2: Yes

2. Has the statistical analysis been performed appropriately and rigorously?

Reviewer #1: Yes

Reviewer #2: Yes

3. Have the authors made all data underlying the findings in their manuscript fully available?

The [PLOS Data policy](http://www.plosone.org/static/policies.action#sharing) requires authors to make all data underlying the findings described in their manuscript fully available without restriction, with rare exception (please refer to the Data Availability Statement in the manuscript PDF file). The data should be provided as part of the manuscript or its supporting information, or deposited to a public repository. For example, in addition to summary statistics, the data points behind means, medians and variance measures should be available. If there are restrictions on publicly sharing data—e.g. participant privacy or use of data from a third party—those must be specified.

Reviewer #1: Yes

Reviewer #2: No

4. Is the manuscript presented in an intelligible fashion and written in standard English?

PLOS ONE does not copyedit accepted manuscripts, so the language in submitted articles must be clear, correct, and unambiguous. Any typographical or grammatical errors should be corrected at revision, so please note any specific errors here.

Reviewer #1: No

Reviewer #2: No

5. Review Comments to the Author

Please use the space provided to explain your answers to the questions above. You may also include additional comments for the author, including concerns about dual publication, research ethics, or publication ethics. (Please upload your review as an attachment if it exceeds 20,000 characters)

Reviewer #1: 1) Title needs to be changed to include “the assessment of psychometric properties __)

Authors: This suggestion was executed
2) PCQ is not introduced, This needs to be introduced before using.

Authors: This suggestion was executed

3) Background: does not highlight the magnitude of the problem, especially among Iranian population. Some factual information stated in this section is not supported by references.

Authors: Changes applied. Several references have been added
4) How 205 mothers were selected for the validation part of the study is not stated. What were the inclusion/exclusion criteria?

Authors: inclusion/exclusion criteria are added in method section.

The KMO index measures the adequacy of the sample size for conducting factor analysis. A higher KMO value indicates that the sample is appropriate for identifying underlying relationships among variables. This is essential for ensuring that the factor analysis yields reliable and valid results.

The KMO index helps determine whether the data is suitable for factor analysis by evaluating the correlations between variables. A KMO value closer to 1 suggests that the data is suitable for factor analysis, while a value below 0.5 indicates that the data may not be suitable. In the present study Kaiser-Meyer-Olkin Measure of Sampling Adequacy was: 0. 777.

The KMO index is often used alongside Bartlett's Test of Sphericity, which tests the hypothesis that the correlation matrix is an identity matrix. Together, these tests provide a comprehensive assessment of whether the data is appropriate for factor analysis. Based on our results KMO P- value = 0.000.

5) Some tables are huge and the journal is unable to include then in the manuscript. Authors and editors need to think of a way to make this information available outside the manuscript.

Authors: We have presented the results and tables for easier understanding for the readers. Some tables have been moved to the appendix section based on the opinion of the referees and the respected editor.

6) It is unclear whether the final version of the questionnaire was made in English language or local language. Also whether authors allow access to the full questionnaire.

Authors: Final version of the questionnaire was made in Persian and translated into English in the current article. Researchers will allow the use of the questionnaire to researchers if they comply with ethics in research.

7) Although the manuscript is well written, there are some grammatical errors. eg. use of present and past tense.

Authors: The article was referred to someone who was fluent in English and spelling and grammar corrections were made as much as possible.

Reviewer #2: 1. The manuscript require professional proof reading.
2. Items' generation procedure must be explained precisely both in the "methodology" and "results" sections.

Authors: All steps in generating the items are listed in the Methods and Results, step by step. Some in the text and others in tables.

3. Detailed information is needed in the result section to reflect how the number of identified items reduced from 41 to 34 as stated in page 28.

Authors: The phases involved in the reduction of items, from the creation of the initial item pool to the completion of the final questionnaire version are presented in Table 6.

4. The study limitations were written very superficially and must include a comprehensive list of methodological limitations that might pose bias on the study findings.

Authors: Strengths and limitations have been summarized and rewritten based on the reviewers' comments.

6. PLOS authors have the option to publish the peer review history of their article ([what does this mean?](https://journals.plos.org/plosone/s/editorial-and-peer-review-process#loc-peer-review-history)). If published, this will include your full peer review and any attached files.

If you choose “no”, your identity will remain anonymous but your review may still be made public.

**Do you want your identity to be public for this peer review?** For information about this choice, including consent withdrawal, please see our [Privacy Policy](https://www.plos.org/privacy-policy).

Reviewer #1: **Yes:** Sarath Lekamwasam

Reviewer #2: No

[NOTE: If reviewer comments were submitted as an attachment file, they will be attached to this email and accessible via the submission site. Please log into your account, locate the manuscript record, and check for the action link "View Attachments". If this link does not appear, there are no attachment files.]

While revising your submission, please upload your figure files to the Preflight Analysis and Conversion Engine (PACE) digital diagnostic tool, <https://pacev2.apexcovantage.com/>. PACE helps ensure that figures meet PLOS requirements. To use PACE, you must first register as a user. Registration is free. Then, login and navigate to the UPLOAD tab, where you will find detailed instructions on how to use the tool. If you encounter any issues or have any questions when using PACE, please email PLOS at [figures@plos.org](mailto:figures@plos.org). Please note that Supporting Information files do not need this step.
